# Evaluation of PD-L1 Expression in Tumor Tissue of Patients with Lung Carcinoma and Correlation with Clinical and Demographic Data

**DOI:** 10.1155/2016/9839685

**Published:** 2016-09-22

**Authors:** Gustavo Dix Junqueira Pinto, Luciano de Souza Viana, Cristovam Scapulatempo Neto, Sérgio Vicente Serrano

**Affiliations:** ^1^Department of Medical Oncology, Barretos Cancer Hospital, Barretos, SP, Brazil; ^2^Department of Oncology, Marcio Cunha Hospital, Fundação São Francisco Xavier, Ipatinga, MG, Brazil; ^3^Post Graduation Program in Oncology, Barretos Cancer Hospital, Barretos, SP, Brazil; ^4^Pathology, Clinicas Oncologicas Integradas, Rio de Janeiro, RJ, Brazil; ^5^Barretos School of Health Sciences (FACISB), Barretos, SP, Brazil

## Abstract

Lung cancer is the leading world cause of cancer-related death, in both genders, and smoking is the main etiological factor. The discovery of immune checkpoints corroborates the hypothesis that ligands presented in tumors modulate the mechanisms of carcinogenesis and the immune activity of tumor microenvironment. Among the most studied coregulatory molecules, PD-1* (programmed cell death 1)* and its ligand PD-L1* (programmed cell death 1 ligand 1)* are noteworthy. The present study aims to enhance the understanding of the tumor microenvironment of lung cancer patients who underwent surgery, by means of analysis of PD-L1 expression in tumor cells and in intratumoral immune cells (IICs). It was found that PD-L1 expression was more frequent in tumor cells than in IICs. Collective analysis by Tissue Microarray Assay (TMA) for PD-L1 expression in tumor cells and IICs did not reproduce the findings for separate individual analysis of tumor tissues. Patients with past history of smoking were more likely to express PD-L1 in tumor cells than those who never smoked. Patients with past history of smoking were less likely to have PD-L1 positive IICs compared to those who had never smoked. The immunohistochemical expression of PD-L1 in tumor cells and IICs did not correlate with survival.

## 1. Introduction

Lung cancer remains the leading cause of cancer death worldwide, for both men and women. Over half of people diagnosed with lung cancer die within one year of diagnosis and the 5-year survival is less than 18% [1].

In Brazil, according to 2016 estimates of INCA (National Cancer Institute), the incidence rate for tracheal, bronchus, and lung tumors will be 17330 new cases (8.1% of total) for men and 10890 cases (5.3% of total) for women [2].

Despite major advances in the personalized medicine, non-small-cell lung cancer is still related to poor prognosis.

Until recently, non-small-cell lung cancer was considered a nonimmunogenic tumor, but there is now evidence highlighting the integral role played by both inflammatory and immunological responses in lung carcinogenesis [3]. The discovery of immune checkpoints corroborates the hypothesis that ligands presented in tumors modulate the mechanisms of carcinogenesis and the immune activity of tumor microenvironment. New strategies in immunotherapy are targeting immune-modulating mechanisms that help tumor cells defend themselves against the immune system. Immune checkpoints are inhibitory pathways that maintain self-tolerance and protect the peripheral tissues by modulating the immune responses [4].

Recent studies show that tumor cells and antigen presenting cells modify tumor microenvironment through PD-1 receptor activities involving their ligands, PD-L1 and PD-L2 [5, 6]. PD-1 is a type I membrane protein composed of 268 amino acids which belongs to T-cell CD28/B7 family and is encoded by PDCD1 gene [7, 8]. It has an extracellular IgV domain, followed by a transmembrane region, and an intracellular tail, which contains two phosphorylation sites [9, 10] and is expressed on the surface of activated T cells, B cells, and macrophages [10].

Programmed cell death 1 ligand 1 (PD-L1) or B7 homolog 1 (B7-H1) is a type I transmembrane protein encoded by the* CD274* gene [11].

The PD-1 function occurs primarily in peripheral tissues, where the T cells can contact their immunosuppressive ligands PD-L1 (B7-H1) and PD-L2 (B7-DC), which are expressed by tumor cells, stromal cells, or both [12–15]. It has been shown that inhibition of PD-1/PD-L1 interaction can exacerbate in vitro T cell response and mediate antitumor activity in preclinical models [14, 16].

More than that, PD-L1 expression may also vary according to different tumor microenvironments [17] and even to clinical and demographic data.

One recent study showed that PD-L1 expression was not associated with gender, histology, differentiation status, or lymph node metastasis. However, PD-L1 expression was increased in stage III NSCLC compared with stage I/II [18].

Other studies showed that PD-L1 expression was significantly associated with smoking [19–21], gender, higher tumor grade, advanced T status, advanced N status, advanced stage [19], and histology [22].

The role of PD-L1 expression neither as a prognostic nor as a predictive factor is controversial, being suggested in several studies [8, 23, 24]. Other studies do not confirm these findings [25, 26].

Indeed, recently, the KEYNOTE-001 trial of pembrolizumab (a humanized antibody that targets the programmed cell death 1 receptor) for advanced NSCLC showed a significantly favourable survival in patients with that PD-L1 expression greater than 50% in comparison to those with expression lower than 50% [27].

PD-L1 seems to be a mutable biomarker, with variable expression patterns related to heterogeneity in different areas within primary or metastatic lesions.

Histopathological material aging or the interval between tissue collection and treatment may also influence PD-L1 expression [28–30].

Positivity criteria for PD-L1 expression (machinery, antibodies, and cutoff) are not standardized yet and that certainly may contribute for all these discrepancies.

This study attempts to elucidate PD-L1 expression in lung tumor microenvironment. Possibly it may help to understand different clinical outcomes in lung cancer and establish more effective therapeutic measures.

## 2. Materials and Methods

### 2.1. Study Population

Tumor tissue samples and data were obtained from 177 cases. These patients underwent surgical resection of primary lung cancer between 2003 and 2014 at Barretos Cancer Hospital, Brazil. H&E stained sections were reviewed by a pathologist (CSN) and histological subtyping was assessed using the current World Health Organisation 2015 classification. Staging was undertaken according to the 7th edition AJCC tumor, node, metastasis (TNM) classification. The ethical use of human tissue for research was approved by the Institutional Review Board, and the design of this study followed the principles of the Declaration of Helsinki and also complied with the principles of good clinical practice.

### 2.2. Inclusion Criteria

Inclusion criteria include patients older than 18 years of both genders, diagnosed with non-small-cell lung cancer (adenocarcinoma, adenosquamous carcinoma, and squamous cell carcinoma), who underwent pneumonectomy, lobectomy, segmentectomy, or nodulectomy with available slides and paraffin blocks for histopathological analysis in the Pathology Department of Barretos Cancer Hospital.

### 2.3. Exclusion Criteria

Exclusion criteria include inappropriate histopathological material by either poor quality or shortage due to its use in other studies and patients with a current diagnosis of another primary malignancy in any location of the body other than nonmelanoma skin cancer or in situ carcinoma of the cervix.

### 2.4. PD-L1 Expression

PD-L1 immunohistochemistry membranous expression was assessed in TMA (Tissue Microarray Assay) with 2 cores of 1 mm in diameter of each tumor and also the expression was evaluated in whole tissue section of the same tumors (individual analysis).

### 2.5. TMA Blocks and Slides Construction

TMA blocks were made with MTA I (Manual Tissue Arrayer) device (Estigen, Tartu, Estonia), according to manufacturer's specifications. TMA and all the paraffin blocks were sectioned with 4*μ* thick and the sections were transferred to positively charged glass slides.

At least one section per paraffin block was stained with hematoxylin-eosin in order to confirm the histology and to certify of tumor tissue availability in the slides by a pathologist and another slide was submitted to PD-L1 immunohistochemistry test.

### 2.6. Immunohistochemistry

Four micra thick sections (TMA and whole tissue sections) were dewaxed at 80°C for 20 minutes and then were transferred to Ventana BenchMark Ultra Autostainer (Ventana, Tucson, AZ, USA). Antigen retrieval was performed using Ventana Cell Conditioning solution 1 (pH: 8.5) for 64 minutes at 97°C. Anti-PD-L1 antibody (ab58810, Abcam, Cambridge, MA, USA) at titration of 1 : 25 was incubated for 60 minutes at 36°C. The detection of antigen antibody reaction was performed using UltraView DAB Universal Detection kit (Ventana, Tucson, AZ, USA). Strong membranous trophoblastic staining in the placenta was used as positive control. Tissue expression of PD-L1 was categorized dichotomically into negative (<5% membranous tumor cells expression) or positive (≥5% membranous tumor cells expression) [31] and also in groups according to the percentage of stained cells and staining intensity for a better sample description. The PD-L1 positive intratumoral immune cells (IICs) were graded as absent, 1+ (present until 10% of tumor surface), 2+ (present in 11–50% of tumor extension), and 3+ (present in more than 50% of tumor surface). Also, for statistical purposes PD-L1 expression was graded as present and absent.

### 2.7. Statistics

Data were described on the average, standard deviation, minimum, maximum, and quartiles for quantitative variables and frequency tables for qualitative variables. The agreement between the collective analysis technique or TMA (tissue microarray) and individual analysis was done using the Kappa coefficient to assess the reproducibility.

The correlation between clinical and demographic characteristics and PD-L1 expression in tumor cells and IICs was determined using the chi-square test (or Fisher's exact test) for the qualitative characteristics. To check the overall association between covariates and PD-L1 marker, those that had lower *p* value than 0.2 in the previous test were selected and subsequently adjusted into a logistics multiple regression model. For modeling, one feature (variable) was removed each time by prioritizing those with higher *p* value, up to a set of significant variables.

For overall survival, the time between diagnosis and death for any reason (which is the event of interest) or last objective information of individuals who have not died yet was considered. To compare each feature and check the list of features with survival (one at each time), simple Cox regression was used. For joint evaluation between variables, those with *p* value less than 0.2 were selected in simple analysis and adjusted in multiple Cox regression model. Then, modeling was continued as the same description in the logistic regression. To estimate the overall survival curve for PD-L1 expression the Kaplan-Meier method was used.

For survival analysis only overall survival was considered, because it is a retrospective study and there was no standardization in the follow-up of these patients in the past. In this study we considered the statistical significance of 0.05 and SPSS 21.0 software was used for statistical analysis.

## 3. Results 

### 3.1. Demographic Data

In regard to demographic characteristics, as seen in Table 1, most patients were over 60 years old (96 cases; 54.2%), male (111 cases; 62.7%), white (140 cases; 79.1%), ECOG (Eastern Cooperative Oncology Group performance status) 0 (86 cases; 48.6%), with reported history of smoking (79 cases; 44.6%), and with no history of alcohol abuse (96 cases; 54.2%).

### 3.2. Clinical Data

The most common histological type was adenocarcinoma (115 cases, 65%) with II degree of differentiation (81 cases; 45.8%). Most of tumors had TNM Classification of Malignant Tumours lower than III. Stages I and II were observed in 62 patients (35%) and 53 patients (29.9%), respectively. Lobectomy was performed in 147 cases (83.1%). Adjuvant chemotherapy was performed in 34 cases (19.2%) (Table 2).

### 3.3. *PD-L1 Expression *in TMA and Whole Tissue Section

PD-L1 was expressed in tumor cells and in IICs. Tumor cells had coarse chromatin, increased nuclei with irregular nuclear membrane, and abundant cytoplasm with ill-defined borders. However, intratumoral immune cells had loose chromatin, smaller fold nuclei without atypia, and variable cytoplasm, sometimes with dendritic expansions (Table 3).

**Figure 1:**
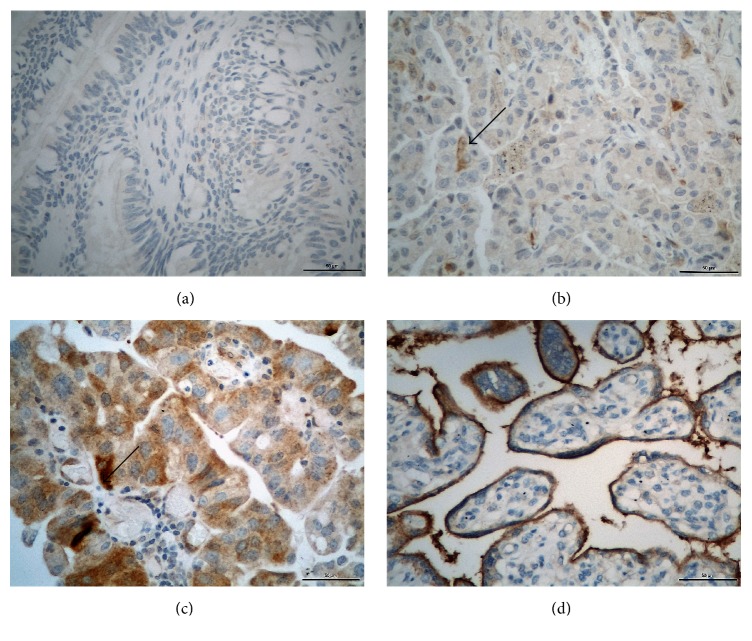
Photomicrographs of PD-L1 immunohistochemical expression in lung cancer tumor cells (magnification/scale bar: 400x/50*μ*). (a) Absence of PD-L1 expression; (b) negative PD-L1 expression (<5% of stained cells); (c) positive PD-L1 expression (≥5% stained cells); (d) Positive Control with placental tissue. Arrows show positive staining cells.

**Figure 2:**
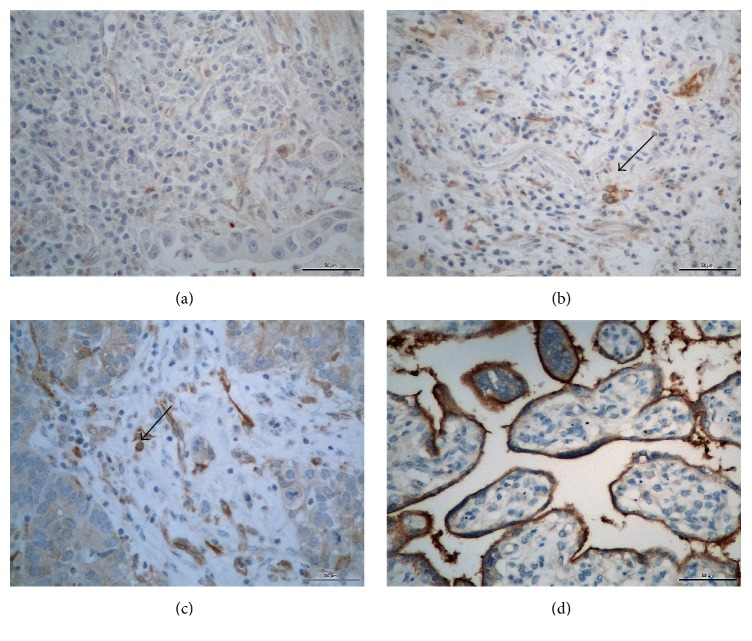
Photomicrographs of PD-L1 immunohistochemical expression in IICs in non-small-cells lung cancer (magnification/scale bar: 400x/50*μ*). (a) Absence of PD-L1 expression; (b) focal PD-L1 expression (<5% of tumor surface); (c) diffuse PD-L1 expression (>5% of tumor surface). Observe the dendritic cytoplasm of some cells; (d) Positive Control. Arrows show positive staining cells.

In Figures 1 and 2, there are photomicrographs of PD-L1 immunohistochemical expression in conventional histological slides in tumor cells and antigen presenting cells, respectively.

**Figure 3:**
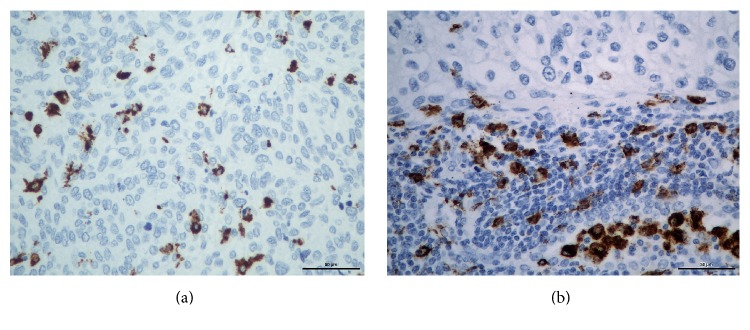
Photomicrographs of CD-68 immunohistochemical expression in IICs in non-small-cells lung cancer (magnification/scale bar: 400x/50*μ*). (a) Isolated IICs in a tumor area. (b) Positive CD-68 expression in a cluster of inflammatory cells.

In Figure 3 there are photomicrographs of CD-68 immunohistochemical expression in IICs of non-small-cells lung cancer.

Table 4 contains the results of PD-L1 expression by immunohistochemistry in TMA slides (collective analysis). Positive expression is observed in 58 cases (32.8%) in tumor cells and in 35 cases (19.8%) of IICs, that is, higher expression in tumor cells.

### 3.4. Immunohistochemical Agreement of PD-L1 Expression in Individual Analysis (Whole Section) versus Collective Analysis (TMA)

As noted in Table 5, there was a fair agreement with Kappa index of 0.307 (*p* < 0.001) when the immunohistochemical expression of PD-L1 in tumor cells in conventional histological slides (individual analysis) and TMA slides (collective analysis) was evaluated. Similar results were observed with PD-L1 expression in IICs in conventional histological slides (individual analysis) and IICs slides (collective analysis) with Kappa index of 0.328 (*p* < 0.001).

Once the immunohistochemical expression of PD-L1 was not reproducible between TMA and whole tissue sections, all the analyses (univariate and multivariate) of correlation between PD-L1 expression and covariates (clinical data and demographic and other biomarkers) considered PD-L1 expression in whole tissue section.

### 3.5. Association of PD-L1 Expression with Demographic Data: Univariate Analysis

As noted in Table 6, patients whose tumors showed positive PD-L1 expression in tumor cells were mostly former smokers (36 cases; 55.4%) or active smokers (20 cases; 30.8%) with *p* value = 0.044.

As seen in Table 7, the correlation between PD-L1 expression in IICs and demographic data was not statistically significant.

### 3.6. Correlation of PD-L1 Expression with Clinical Data

As seen in Table 8, the correlation between PD-L1 expression in tumor cells and clinical data was not statistically significant.

According to Table 9, among patients whose tumors had positive PD-L1 expression in IICs, most of them presented histologic type adenocarcinoma (35 cases; 81.4%) with statistical significance (*p* value = 0.022).

### 3.7. Correlation of PD-L1 Expression with Clinical and Demographic Data: Multivariate Analysis

Patients with smoking history were more likely to present positive PD-L1 expression in tumor cells when compared to patients who had never smoked (OR = 3.356; 95% CI 1.368 to 8.230; *p* value = 0.008) (Table 10).

After logistic regression, smoking was the only variable that remained significant related to PD-L1 expression in antigen presenting cells.

Patients with smoking history were less likely to present tumors with positive PD-L1 expression in IICs when compared to patients who had never smoked (OR = 0.383; 95% confidence interval from 0.162 to 0.908; and *p* value = 0.029). The chance of positive PD-L1 expression in intratumoral immune cells was also lower for patients with active smoking when compared to patients who had never smoked but without statistical significance (OR = 0.525; 95% CI 0.212 to 1.297; and *p* value = 0.169) (Table 11).

### 3.8. Survival Curves Analysis according to PD-L1 Expression

**Figure 4:**
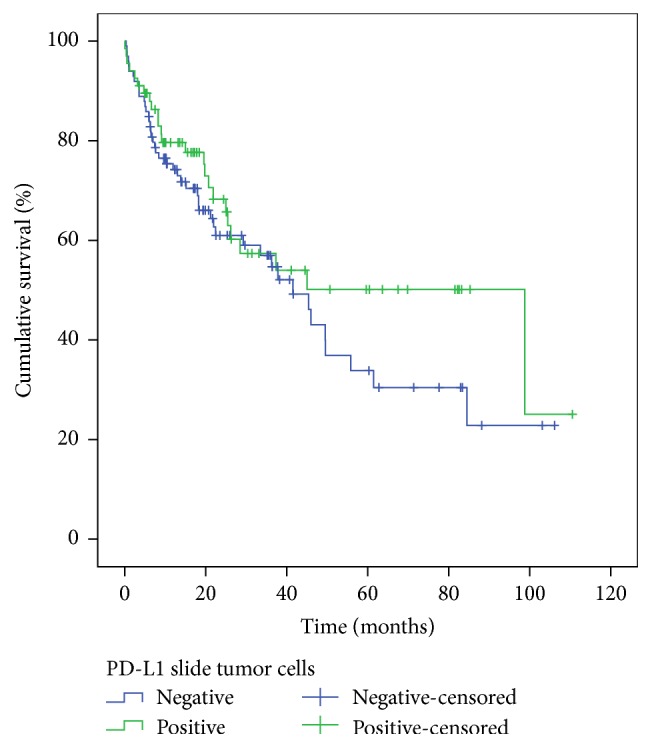
Overall survival according to PD-L1 expression in tumor cells.* x*-axis: time after diagnosis in months.* y*-axis: percentage of patients alive (total of 177 patients).

**Figure 5:**
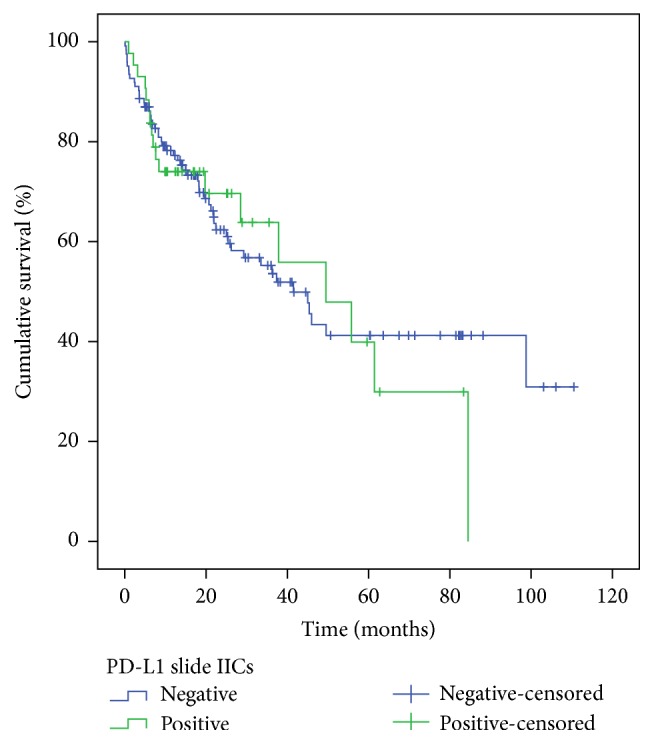
Overall survival according to PD-L1 positivity in IICs.* x*-axis: time after diagnosis in months.* y*-axis: percentage of patients alive (total of 177 patients).

The median overall survival of patients included in this study was 45 months (95% CI 33.23 to 56.83). Considering PD-L1 expression in tumor cells of conventional histological slides (individual analysis), there was a higher median overall survival for patients with tumors that had positive PD-L1 expression; however this difference was not statistically significant: 98.75 months (95% CI 21.26 to 176.24) versus 41.51 months (95% CI 30.05 to 52.98) with *p* value = 0.254. Also, there was no statistically significant difference in the median overall survival when comparing PD-L1 expression in IICs: 49.50 months (95% CI 23.37 to 75.64) for patients with positive PD-L1 expression versus 41.51 months (95% CI 29.99 to 53.04) in patients with negative PD-L1 expression with *p* value = 0.795 (Figures 4 and 5).

## 4. Discussion

### 4.1. PD-L1 Expression Findings

According to this study, the frequency of PD-L1 expression in tumor cells was 37.9% of total cases, a little less than a recently published study that showed 53.1% of positivity [32], although the same antibodies and platforms have been used. There is a bias when comparing positivities related to different studies. There are different platforms (DAKO and VENTANA) to evaluate PD-L1 expression and different antibodies (28-8, 22C3, SP263, SP142, and MIH1, among others).

This evaluation can occur in different ways: continuous distribution and proportion of PD-L1 positive cells at any intensity [27], percentage expression (immunohistochemistry score) regardless of its intensity (more than 1% of stained cells: score 1, more than 5% stained cells: score 2, and more than 10% stained cells: score 3) [33], a combined score displaying a percentage for each intensity [34] by degree in membrane and/or cytoplasmic staining (e.g., Aqua Fluorescent Techniques), and protein concentration in Tissue Microarray Assay [35].

There is no standardized cutoff for positivity. Some studies have used 1%, 5%, 50%, or more to consider positive PD-L1 expression [36].

Therefore, it becomes extremely difficult to establish any relationship between studies as a consequence of no standard positivity criteria for PD-L1 expression, which involves the whole process, from chemical reactions machinery to dilutions of reactants and its reading interpretations. As seen in Table 12, there are different studies with different cutoffs for PD-L1 expression positivity.

Another important issue to be discussed is the feasibility of PD-L1 biomarker in order to be measured and analyzed in histopathological slides. Garon et al. noted PD-L1 deterioration in tumor samples cuts more than 6 months before staining [27]. According to Calles et al., there is deterioration of PD-L1 in blocks of more than three years [21]. In this study, staining was done less than a month after the cuts and due to this possible deterioration a lower cutoff of 5% was chosen.

A recent study showed that tumor microenvironment cells (including tumor cells, lymphocytes, and antigen presenting cells) do not express PD-L1 in a uniform way [36]. Smyth et al. argue that a successful cancer treatment must be precisely based on the stratification of tumor microenvironment and besides that it should not be restricted only to immune checkpoints but also to potentially involved cell types [42], as they are best studied and characterized.

Tumor microenvironment has a wide range of different cell types mainly represented by myeloid-derived suppressor cells, macrophages, dendritic cells, and T lymphocytes beyond tumor cells [43]. There is clearly a consensus that tumor microenvironment is a complex and dynamic structure so far as its presentation may vary for different time periods. The emergence of tumor cells leads to drastic changes that initiate immunity chronologically with an intrinsic induction of PD-L1 expression followed by adaptive immune tolerance and resistance to quantitative variations of various cell types, previously described [17].

### 4.2. Immunohistochemical Agreement between PD-L1 Expression in Individual Analysis and Collective Analysis (TMA)

This study analysis showed a fair (low) agreement between the two methods of immunohistochemistry (individual and collective), for PD-L1 expression evaluation in lung tumors. The medical literature has pointed to the importance that lung tumor heterogeneity may have regarding PD-L1 expression in small biopsies when compared to surgical specimens.

Small isolated samples from a particular tumor region gathered by biopsy or punches for TMA construction, for example, cannot represent PD-L1 expression for the tumor as a whole. This expression cannot be generalized, because of its intrinsic regional variability. Immunohistochemistry assessment of slides covering a tumor in its largest possible area, with the largest represented diameters, seems to provide a more representative description of real PD-L1 expression in lung tumors.

### 4.3. Correlation of PD-L1 Expression with Clinical and Demographic Data

In this study, tumors of patients with smoking history were more likely to positively express PD-L1 in tumor cells and they were also more likely to not express this biomarker in IICs. Although the influence of smoking in PD-L1 expression in tumor cells [21] has already been described, this is the first time that the possible influence of smoking in PD-L1 expression related to IICs is shown. Active smokers and former smokers were compared to nonsmokers (reference category after logistic regression). A possible explanation for the inverse relationship between smoking and PD-L1 expression in tumor cells versus IICs is the association between inflammatory proteins and such cell types with still unknown biological effects.

It is worth noting that changes in PD-L1 expression can also occur related to previous exposure to chemotherapy, a fact already demonstrated in urothelial carcinomas and lung cancer [44, 45]. The small number of patients undergoing neoadjuvant chemotherapy prevented this analysis in the present study.

### 4.4. Survival Curves Analysis according to PD-L1 Expression

There was no statistical significance related to PD-L1 expression and overall survival as previously described. Other studies show apparently not to be a predictive or prognostic value regarding PP-L1 expression [27, 46, 47]. However, in a recently published meta-analysis, after analyzing five trials with 877 patients with non-small-cell lung cancer, Zhou et al. concluded that PD-L1 expression may be related to a worse prognosis [48]. Nonetheless, only one of these studies included western patients outside China. Variations in the choice of different cutoffs for expression of positivity and the type of tissue sample may have contributed to discrepant results.

In summary, this study shows that, in a population of patients with non-small-cell lung cancer, the pattern of PD-L1 expression was heterogeneous, represented by two distinct cell types: the tumor cell (higher frequency) and IICs (lowest frequency). Positive PD-L1 expression was observed in 37.9% of tumor cells and in IICs of 24.3% of tumors.

TMA techniques (collective analysis) and conventional histological slides (individual analysis) showed fair agreement when they evaluated the immunoreactivity of PD-L1 expression in tumor cells (Kappa = 0.307) and in antigen presenting cells (Kappa = 0.328) of patients with non-small-cell lung cancer, *p* value < 0.001, proving that TMA is not an adequate method to evaluate PD-L1 expression.

Former smokers had a higher PD-L1 expression in tumor cells when compared to those who had never smoked. In contrast, former smokers had a lower PD-L1 expression in IICs when compared to those who had never smoked. There was also no correlation between patterns of PD-L1 expression and survival in this population.

## Figures and Tables

**Table 1:** Demographic characteristics.

Variable	Category	*N*	(%)
Age	<60 years	81	45.8
	>60 years	96	54.2

Sex	Female	66	37.3
	Male	111	62.7

Ethnicity	White	140	79.1
	Non-white	32	18.1
	Ignored	5	2.8

ECOG	0	86	48.6
	1	46	26
	Other	6	3.38
	Ignored	39	22.02

Smoking	Absent	39	22
	Active	57	32.2
	Past	79	44.6
	Ignored	2	1.1

Alcohol consumption	No	96	54.2
	Active	45	25.4
	Past	18	10.2
	Ignored	18	10.2

ECOG: Eastern Cooperative Oncology Group performance status.

**Table 2:** Clinical data.

Variable	Category	*N*	(%)
Histological type	Adenocarcinoma	115	65
	Squamous cell carcinoma	57	32.2
	Other	5	2.8

Tumor grade	Grade I	5	2.8
	Grade II	81	45.8
	Grade III	50	28.2
	Ignored	41	23.2

TNM staging	I	62	35
	II	53	29.9
	III	43	24.3
	IV	19	10.7

Surgery type	Nodule extraction	4	2.3
	Segmentectomy	12	6.8
	Lobectomy	147	83.1
	Pneumonectomy	14	7.9

Systemic treatment	No	121	68.4
	Yes	55	31
	Neoadjuvant chemotherapy	21	11.9
	Adjuvant chemotherapy	34	19.2
	Chemotherapy + radiotherapy	3	1.7
	Ignored	1	0.6

Adjuvant radiotherapy	Yes	23	13
	No	154	87

TNM staging: TNM Classification of Malignant Tumours.

**Table 3:** PD-L1 expression in conventional histological slides (individual analysis).

Cell type	Evaluation method	Graduation	*N*	%
Tumor cell	Intensity	Absence	97	54.8
		Weak	52	29.3
		Moderate	16	9
		Strong	1	0.6
		Missing	11	6.2

Tumor cell	Percentage (3 categories)	Absence	97	54.8
		Up to 10%	6	3.4
		From 11% to 50%	33	18.6
		More than 50%	30	16.9
		Ignored	11	6.2

Tumor cell	Percentage (2 categories)	Positive	67	37.9
		Negative	99	55.9
		Ignored	11	6.2

Intratumoral immune cells	Intensity	Absence	123	69.5
		Weak	25	14.1
		Moderate	15	8.5
		Strong	3	1.7
		Ignored	11	6.2

Intratumoral immune cells	Percentage (3 categories)	Absence	123	69.5
		Up to 10%	3	1.7
		From 11% to 50%	15	8.5
		More than 50%	25	14.1
		Ignored	11	6.2

Intratumoral immune cells	Percentage (2 categories)	Positive	43	24.3
		Negative	123	69.5
		Ignored	11	6.2

Positive: more than 5% stained cells.

Negative: less than 5% stained cells.

Ignored: staining problems preventing classification into positive or negative.

**Table 4:** PD-L1 expression in TMA slides (collective analysis).

Cell type	Evaluation method	Graduation	*N*	%
Tumor cell	Intensity	Absence	99	55.9
		Weak	38	21.5
		Moderate	16	9
		Strong	4	2.3
		Ignored	20	11.3

Tumor cell	Percentage (3 categories)	Absence	99	55.9
		Up to 10%	0	0
		From 11% to 50%	1	0.6
		More than 50%	57	32.2
		Ignored	20	11.3

Tumor cell	Percentage (2 categories)	Positive	58	32.8
		Negative	99	55.9
		Ignored	20	11.3

Intratumoral immune cells	Intensity	Absence	122	68.9
		Weak	24	13.6
		Moderate	6	3.4
		Strong	5	2.8
		Ignored	20	11.3

Intratumoral immune cells	Percentage (3 categories)	Absence	122	68.9
		Up to 10%	4	2.3
		From 11% to 50%	5	2.8
		More than 50%	26	14.7
		Ignored	20	11.3

Intratumoral immune cells	Percentage (2 categories)	Positive	35	19.8
		Negative	122	68.9
		Ignored	20	11.3

Positive: more than 5% stained cells.

Negative: less than 5% stained cells.

Missing: staining problems preventing classification into positive or negative.

**Table 5:** Immunohistochemical agreement of PD-L1 expression in individual analysis versus collective analysis considering cell type.

Cell type	Kappa	Standard error	*p* value
IICs	0.328	0.089	<0.001
Tumor cells	0.307	0.079	<0.001

**Table 6:** PD-L1 expression in tumor cells and demographic data.

Variable	Categories	Negative		Positive		*p* value
		*N*	(%)	*N*	(%)	
Age	<60	43	43.4	30	44.8	0.864
	>60	56	56.6	37	55.2	

Gender	Female	39	39.4	21	31.3	0.289
	Male	60	60.6	46	68.7	

Race	Non-white	18	18.8	11	16.7	0.734
	White	78	81.2	55	83.3	

ECOG	0	48	62.3	31	70.5	0.367
	1	29	37.7	13	29.5	

Smoking	No	29	29.3	9	13.8	0.044
	Active	31	31.3	20	30.8	
	Former	39	39.4	36	55.4	

Alcohol consumption	No	57	63.3	34	58.6	0.312
	Active	25	27.8	14	24.1	
	Former	8	8.9	10	17.2	

ECOG: Eastern Cooperative Oncology Group performance status.

**Table 7:** PD-L1 expression in IICs and demographic data.

Variable	Categories	Negative		Positive		*p* value
		*N*	(%)	*N*	(%)	
Age	<60	50	40.7	23	53.5	0.144
	>60	73	59.3	20	46.5	

Gender	Female	41	33.3	19	44.2	0.202
	Male	82	66.7	24	55.8	

Race	Non-white	21	17.6	8	18.6	0.888
	White	98	82.4	35	81.4	

ECOG	0	57	64.8	22	66.7	0.845
	1	31	35.2	11	33.3	

Smoking	No	23	19	15	34.9	0.084
	Active	38	31.4	13	30.2	
	Former	60	49.6	15	34.9	

Alcohol consumption	No	67	60.9	24	63.2	0.934
	Active	29	26.4	10	26.3	
	Former	14	12.7	4	10.5	

ECOG: Eastern Cooperative Oncology Group performance status.

**Table 8:** PD-L1 expression in tumor cells and clinical data.

Variable	Categories	Negative		Positive		*p* value
		*N*	(%)	*N*	(%)	
Histological type	Adenocarcinoma	68	68.7	40	59.7	0.412
	Squamous cell carcinoma	28	28.3	25	37.3	
	Other	3	3	2	3	

Tumor grade	I and II	50	62.5	31	64.6	0.813
	III	30	37.5	17	35.4	

TNM staging	I	36	36.4	23	34.3	0.756
	II	27	27.3	23	34.3	
	III	26	26.3	14	20.9	
	IV	10	10.1	7	10.4	

Neoadjuvant systemic treatment	No	86	86.9	61	91	0.407
	Yes	13	13.1	6	9	

TNM staging: TNM Classification of Malignant Tumours.

**Table 9:** PD-L1 expression in antigen presenting cells and clinical data.

Variable	Categories	Negative		Positive		*p* value
		*N*	(%)	*N*	(%)	
Histological type	Adenocarcinoma	73	59.3	35	81.4	0.022
	Squamous cell carcinoma	46	37.4	7	16.3	
	Others	4	3.3	1	2.3	

Tumor grade	I and II	61	59.8	20	76.9	0.106
	III	41	40.2	6	23.1	

TNM staging	I	42	34.1	17	39.5	0.416
	II	41	33.3	9	20.9	
	III	27	22	13	30.2	
	IV	13	10.6	4	9.3	

Neoadjuvant systemic treatment	No	111	90.2	36	8.7	0.270
	Yes	12	9.8	7	16.3	

TNM staging: TNM Classification of Malignant Tumours.

**Table 10:** Multivariate analysis: PD-L1 expression in tumor cells.

Variable	Categories	OR	95% CI		*p* value
			LI	LS	
Smoking	Never	1			0.03
	Active	2.236	0.864	5.79	0.097
	Past	3.356	1.368	8.23	0.008

**Table 11:** Multivariate analysis: PD-L1 positive IICs.

Variable	Categories	OR	95% CI	*p* value
Smoking	Never	1		0.090
	Active	0.525	0.212–1.297	0.162
	Past	0.383	0.162–0.908	0.029
	Constant	0.652		0.198

**Table 12:** Immunohistochemistry for PD-L1 in clinical studies.

PD-L1 antibody	Cutoff for positivity of PD-L1 expression in tumor cell membrane	Percentage of tumor samples expressing PD-L1	Study reference
28-8	5%	49%	Grosso et al., JCO, 2013 [37]
R&D B7-H1	NR	52%	Gatalica et al., CEBP, 2014 [38]
MIH1	>10%	50%	Konishi et al., CCR, 2004 [39]
5H1	>1% versus >5% versus high score	21% (only CEC)	Marti et al., CCR, 2014 [30]
SP142	5%	60%	
NR	1%	50%	Sun et al., JCO, 2014 [40]
22C3	≥50%	25%	Garon et al., NEJM, 2015 [27]
28-8	≥1%, ≥5%, ≥10%	53%, 36%, 25% (only CEC)	Brahmer et al., NEJM, 2015 [33]
SP142	≥1%, ≥5%, ≥10%	68%, 37%, 16%	
SP142	≥1%, ≥5%, ≥10%	56%, 28%, 13%	Herbst et al., Nature, 2014 [41]
